# Diabetes mellitus, koronare Herzkrankheit und Herzinsuffizienz (Update 2023)

**DOI:** 10.1007/s00508-023-02183-7

**Published:** 2023-04-20

**Authors:** Martin Clodi, Christoph H. Saely, Friedrich Hoppichler, Michael Resl, Clemens Steinwender, Harald Stingl, Thomas C. Wascher, Yvonne Winhofer, Harald Sourij

**Affiliations:** 1 Abteilung für Innere Medizin, Konventhospital der Barmherzigen Brüder Linz, Linz, Österreich; 2 Klinisches Forschungsinstitut für Kardiometabolische Forschung, Johannes Kepler Universität Linz, Altenberger Straße 69, 4040 Linz, Österreich; 3 VIVIT Institut Feldkirch, Feldkirch, Österreich; 4 Private Universität im Fürstentum Liechtenstein, Triesen, Liechtenstein; 5 Abteilung für Innere Medizin I, Akademisches Lehrkrankenhaus Feldkirch, Feldkirch, Österreich; 6 Abteilung für Innere Medizin, Krankenhaus der Barmherzigen Brüder Salzburg, Salzburg, Österreich; 7 Klinik für Kardiologie und internistische Intensivmedizin, Kepler Universitätsklinikum Linz, Linz, Österreich; 8 Karl-Landsteiner-Universität für Gesundheitswissenschaften, Krems an der Donau, Krems, Österreich; 9 Abteilung für Innere Medizin, Landesklinikum Baden, Baden, Österreich; 10 1. Medizinische Abteilung, Mein Hanusch-Krankenhaus, Wien, Österreich; 11 Klinische Abteilung für Endokrinologie und Stoffwechsel, Universitätsklinik für Innere Medizin III, Medizinische Universität Wien, Wien, Österreich; 12 Klinische Abteilung für Endokrinologie und Diabetologie, Universitätsklinik für Innere Medizin, Medizinische Universität Graz, Graz, Österreich

**Keywords:** Diabetes mellitus, Diabetische Herzinsuffizienz, Koronare Herzkrankheit, Herzinsuffizienz, Screening, Diabetes mellitus, Heart failure in diabetes, Coronary artery disease, Heart failure, Screening

## Abstract

Die Zusammenhänge zwischen Diabetes mellitus, koronarer Herzkrankheit und Herzinsuffizienz sind wechselseitig. Bei Erstmanifestation einer koronaren Herzkrankheit sollte aktiv auf Diabetes mellitus gescreent werden, während bei an Diabetes mellitus erkrankten Patient:innen die kardiovaskuläre Risikostratifizierung immer in Zusammenschau sämtlicher Risikofaktoren, Biomarker und dem klinischen Befinden des Patient:innen durchgeführt werden sollte. Eine bereits bekannte kardiovaskuläre Erkrankung wie auch das Vorliegen zahlreicher Risikofaktoren stellen wesentliche Kriterien für die Auswahl der individuellen Therapiestrategien dar.

## Abstract (en)

Diabetes mellitus, cardiovascular disease and heart failure are interacting dynamically. Patients being diagnosed with cardiovascular disease should be screened for diabetes mellitus. Enhanced cardiovascular risk stratification based on biomarkers, symptoms and classical risk factors should be performed in patients with preexisting diabetes mellitus. In patients with previously diagnosed arterosclerotic cardiovascular disease an agent proven to reduce major adverse cardiovascular events or cardiovascular mortality is recommended.

Kardiovaskuläre Erkrankungen stellen die häufigste Ursache für Morbidität und Mortalität bei an Diabetes mellitus Typ 2 erkrankten Patient:innen dar. Neben den mit Diabetes mellitus sehr häufig assoziierten Risikofaktoren wie arterielle Hypertonie, Dyslipidämie und Bewegungsmangel, deren dynamische Wechselwirkung individuell unterschiedlich ist, nimmt Diabetes mellitus als unabhängiger Risikofaktor für kardiovaskuläre Erkrankungen eine maßgebliche Rolle ein.

Die Interaktionen zwischen Diabetes mellitus und kardiovaskulären Erkrankungen sind wechselseitig. Daten aus Österreich belegen die hohe Inzidenz von Glukosestoffwechselstörungen bei Patient:innen, die sich einer Koronarangiographie unterziehen. Von 1040 Patient:innen hatten insgesamt 60 % der Patient:innen einen abnormen Glukosestoffwechsel. Umgekehrt unterstreichen diese Ergebnisse die hohe Inzidenz der koronaren Herzkrankheit im Stadium der gestörten Glukosetoleranz. Unabhängig von den Risikofaktoren lässt sich eine Dosis- Wirkungsbeziehung zwischen gestörter Glukosetoleranz und koronarer Herzkrankheit nachweisen [1].

Stumme myokardiale Ischämien treten bei Diabetes mellitus gehäuft auf und sind in Kombination mit Dyslipidämie, Hypertonie, Rauchen, positiver Familienanamnese, Mikro- oder Makroalbuminurie signifikant mit dem Ausprägungsgrad der Koronarsklerose assoziiert [2].

Auch als Folge der koronaren Herzkrankheit ist die Inzidenz der Herzinsuffizienz bei Diabetes mellitus deutlich gesteigert [3]. Ein Anstieg des HbA_1c_ um 1 % ist mit einem um 8 % erhöhten Risiko für Herzinsuffizienz assoziiert [4]. Das Vorliegen einer Herzinsuffizienz ist mit einer dramatischen Verschlechterung der Prognose der Patient:innen verbunden [5]. Für die Entstehung einer Herzinsuffizienz bei Diabetes mellitus werden Hypertonie, koronare Herzkrankheit (KHK), kardiovaskuläre autonome diabetische Neuropathie, Hyperglykämie und Hyperinsulinämie als gesicherte Risikofaktoren angesehen [6]. Die kardiovasukuläre autonome diabetische Neuropathie bewirkt eine reduzierte Herzfrequenzvariabilität mit Ruhetachykardie (Vagusläsion) und fixierter Herzfrequenz. Neben Hypertonie, KHK und kardiovaskulärer autonomer diabetischer Neuropathie bewirkt die bestehende Hyperglykämie ebenso bedeutende strukturelle wie auch metabolische Veränderungen im Herzmuskel. Auch bei an Diabetes mellitus Typ 1 erkrankten Patient:innen stellen die Diabetesdauer und das HbA_1c_ besonders wichtige Risikofaktoren für die Entstehung einer diabetischen Herzinsuffizienz dar. Zunehmende Bedeutung erfährt die Herzinsuffizienz mit erhaltener linksventrikulärer Funktion (HFpEF) bei an Diabetes mellitus erkrankten Menschen. Die Diagnose dieser Form der Herzinsuffizienz erfolgt klinisch sowie durch die Echokardiographie (relevante strukturelle Herzerkrankung im Sinne einer linksventrikulären Hypertrophie oder einer Vergrößerung des linken Atriums bzw. eine diastolische Dysfunktion) und durch Bestimmung von NT-proBNP (> 125 pg/ml) oder BNP (> 35 pg/ml) [7].

## Kardiovaskuläre Risikostratifizierung bei Diabetes mellitus

Die in den Leitlinien empfohlene, multifaktorielle Diabetestherapie bewirkt eine wesentliche Reduktion kardiovaskulärer Komplikationen und der Mortalität, welche sich bereits in großen epidemiologischen Analysen dokumentieren lässt [8].

Eine individuelle Evaluierung des aktuell vorliegenden kardiovaskulären Risikos muss regelmäßig, mindestens jedoch einmal jährlich durchgeführt werden.

Generell unterscheidet sich das Vorgehen zwischen symptomatischen und asymptomatischen Patienten.

Bei asymptomatischen Patienten, ohne für koronare Herzkrankheit typische EKG-Veränderungen muss keine weiterführende apparative Abklärung durchgeführt werden.

Diese Empfehlungen basieren maßgeblich auf den Resultaten mehrerer Studien; letztlich ist bei stabiler asymptomatischer koronarer Herzkrankheit ein interventionelles Vorgehen einer optimierten multifaktoriellen Therapie nicht überlegen [9].

Neben den klassischen Risikofaktoren, welche meist Eingang in die Risikokalkulatoren finden kann mit Hilfe der Albuminurie und des NT-proBNP (N-terminales-proB-Typ natriuretisches Peptid) eine erweiterte Risikostratifizierung durchgeführt werden. Dies ist insbesondere von Bedeutung, da niedrige NT-proBNP Spiegel (NT-proBNP < 125 pg/ml) für die Prognose kardiovaskulärer Ereignisse angewandt werden können [10]. Gerade bei asymptomatischen Patienten kann mit Hilfe eines apparativen Screenings (Myokard-Szintigraphie) kein Überlebensvorteil der Patienten erreicht werden [11].

Dennoch gibt es Hinweise, dass die Durchführung einer Computertomographie der Koronargefäße (CT-Angiographie) bei asymptomatischen an Diabetes mellitus erkrankten Patienten in Erwägung gezogen werden kann und derzeit in diesem Patientenkollektiv die beste Sensitivität unter den nichtinvasiven Untersuchungen hat [12, 13].

Prinzipiell sollte die kardiovaskuläre Risikostratifizierung immer in Zusammenschau sämtlicher Risikofaktoren, dem EKG, Biomarker und dem klinischen Befinden des Patienten durchgeführt werden und bei Symptomen im Sinne einer (zunehmenden) relevanten Belastungsdyspnoe, zunehmende Leistungsverminderung oder bei pektanginösen Beschwerden weiterführende Untersuchungen veranlasst werden.

## Glukosesenkende Therapie bei manifester kardiovaskulärer Erkrankung

Eine bereits bekannte kardiovaskuläre Erkrankung wie auch das Vorliegen zahlreicher Risikofaktoren stellen wesentliche Kriterien für die Auswahl der individuellen Therapie dar.

Im Rahmen der LEADER-Studie bewirkte Liraglutid eine signifikante Senkung des präspezifizierten kardiovaskulären Endpunktes, wobei dies hauptsächlich auf einer signifikanten Reduktion kardiovaskulärer Todesfälle basiert (HR 0,87; CI 0,78–0,97; *p* = 0,01 für Superiority) [14]. Für Semaglutid bestätigt die SUSTAIN-6-Studie ebenfalls positive kardiovaskuläre Effekte. Der primäre Endpunkt trat in der Semaglutid-Gruppe signifikant geringer auf (HR 0,74; 0,58–0,95 *p* < 0,001 für Non-Inferiority; *p* = 0,02 für Superiority), wobei im Besonderen auch nicht tödliche Schlaganfälle in der Semaglutidgruppe signifikant reduziert wurden (HR 0,61; 0,38–0,99; *p* = 0,04) [15]. In der ELIXA-Studie (Lixisenatid) und in der EXCSEL-Studie (Exenatid 1 × wöchentlich) wurde die kardiovaskuläre Sicherheit für diese Substanzen belegt, allerdings konnte kein substanzspezifischer kardiovaskulärer Zusatznutzen gezeigt werden [16, 17].

Empagliflozin bewirkt im Vergleich zu Placebo zusätzlich zur bereits etablierten blutzuckersenkenden Therapie eine signifikante Reduktion des primären Endpunkts (kardiovaskulärer Tod, nicht tödlicher Myokardinfarkt, nicht tödlicher Insult) bei bereits kardiovaskulär kranken Patienten um 14 %, weiters waren die Gesamtsterblichkeit, die kardiovaskulären Sterblichkeit sowie Hospitalisierungen aufgrund von Herzinsuffizienz reduziert. Im Vergleich zu den bisher durchgeführten Endpunktstudien zeigte sich bereits innerhalb weniger Monate ein positiver Effekt von Empagliflozin auf den kombinierten kardiovaskulären Endpunkt [18]. Für Canagliflozin liegt mit der CANVAS-Studie ebenfalls eine positive, prospektive randomisiert kontrollierte Endpunktstudie vor, wobei Canagliflozin eine signifikante Senkung des primären Endpunktes (kardiovaskulärer Tod, nicht tödlicher Myokardinfarkt, nicht tödlicher Insult) bewirken konnte. Dem positiven Resultat steht eine signifikant höhere Amputationsrate vor allem im Fußbereich unter Canagliflozin gegenüber [19]. Die substanzspezifische, kardiovaskuläre Sicherheit von Dapagliflozin wurde in der DECLARE-TIMI 58-Studie an 17.160 Patient:innen untersucht, wobei 10.186 Patient:innen am Beginn der Studie keine kardiovaskuläre Erkrankung aufwiesen. Im Rahmen der primären Ergebnisanalyse konnte die kardiovaskuläre Sicherheit von Dapagliflozin bestätigt werden. Darüber hinaus wurde eine signifikante Reduktion des kombinierten Endpunktes aus kardiovaskulären Todesfällen und Hospitalisationen aufgrund von Herzinsuffizienz registriert (HR 0,83; 0,73–0,95; *p* = 0,005). Dieser Effekt war hauptsächlich durch eine Reduktion der Hospitalisierung durch Herzinsuffizienz getrieben. Die Reduktion von Hospitalisierung wegen Herzinsuffizienz konnte auch in der Gruppe mit multiplen Risikofaktoren (Dyslipidämie, Hypertonie, Rauchen) ohne vorhergehende kardiovaskuläre Erkrankung nachgewiesen werden [20].

Kurz gefasst ließen sich in ACCORD, ADVANCE und VADT keine signifikanten Effekte auf makrovaskuläre Ereignisse durch eine intensive Blutzuckerkontrolle darstellen [21–23], wobei eine effektive Blutzuckersenkung besonders, wenn diese bereits bei kurzer Diabetesdauer etabliert wird, langfristig kardiovaskuläre Komplikationen wesentlich reduzieren dürfte. Wird der Nachbeobachtungszeitraum erweitert, so konnte in der VADT-Studie nach 9,8 Jahren eine signifikante Reduktion kardiovaskulärer Ereignisse dokumentiert werden (HR 0,83; CI 0,70–0,99; *p* = 0,04) [24]. Ähnliche Beobachtungen konnten schon früher in der UKPDS-Studie gemacht werden.

Zudem konnte in den Studien ein Effekt beobachtet werden, der als „glykämisches Gedächtnis/glycemic memory“ bezeichnet wird. Darunter versteht man, dass sich Phasen von guter glykämischer Kontrolle langfristig auf das Risiko für kardiovaskuläre Ereignisse auswirken.

In der 2012 publizierten ORIGIN-Studie hatten trotz der kurzen Diabetesdauer 59 % der Teilnehmer:innen ein vorangegangenes kardiovaskuläres Ereignis wobei 35 % einen vorangegangenen Myokardinfarkt erlitten hatten. Im Rahmen der Nachbeobachtungszeit von 6,2 Jahren zeigte sich kein signifikanter Unterschied hinsichtlich kardiovaskulärer Endpunkte zwischen einer Glargin basierten Basalinsulintherapie im Vergleich zur Standardtherapie [25]. Dies konnte auch für das Insulin Degludec im Vergleich zu Insulin Glargin in der DEVOTE-Studie gezeigt werden. Insuline können somit als kardiovaskulär sicher angesehen werden [26].

Das Kollektiv der PROactive-Studie welches eine Population mit bereits fortgeschrittener kardiovaskulärer Erkrankung umfasst, liefert interessante Daten für die Verordnung von Pioglitazon bei kardiovaskulär Kranken. Insgesamt konnte in der Pioglitazongruppe keine signifikante Reduktion im primären Endpunkt allerdings eine signifikante Reduktion im sekundären Endpunkt (Mortalität, nicht-tödlicher Myokardinfarkt und Insult) erreicht werden [27].

In der 2017 publizierten TOSCA-IT-Studie wurde zusätzlich zur etablierten Monotherapie mit Metformin stratifiziert nach Behandlungszentrum und zugrundeliegender kardiovaskulärer Erkrankung eine Randomisierung (Pioglitazon oder Sulfonylharnstoff) durchgeführt. Letztlich konnte kein signifikanter Unterschied zwischen den unterschiedlichen Therapiestrategien (HR 0,96; 95 % CI 0,74–1,26, *p* = 0,79) dokumentiert werden. Nicht zuletzt schlägt sich diese Erkenntnis in der geringen Ereignisrate 1,5 pro 100 Personenjahre nieder und zeigt, dass bei an Diabetes mellitus erkrankten Menschen, welche im Vergleich zu den substanzspezifischen kardiovaskulären Endpunktstudien der letzten Jahre ein deutlich geringeres kardiovaskuläres Risiko aufweisen, Sulfonylharnstoffe und Pioglitazon hinsichtlich kardiovaskulärer Endpunkte als gleichwertig zu betrachten sind. Wie erwartet führte Pioglitazon zu einer signifikant geringeren Hypoglykämierate (10 % vs 34 % *p* < 0,001) [28].

Weiterhin sollte bei der Festlegung der individuellen Therapiestrategie besonderes Augenmerk auf die Vermeidung von Hypoglykämien gelegt werden, da gerade schwere Hypoglykämien mit einem höheren kardiovaskulären Risiko assoziiert sind [29].

Zusammenfassend stellen die aktuellen Erkenntnisse einen weiteren Schritt hinsichtlich der Individualisierung der Diabetestherapie dar. Bei Patient:innen mit bereits bekannter kardiovaskulärer Grunderkrankung soll eine Substanz mit dokumentierten positiven kardiovaskulären Effekten eingesetzt werden.

## Glukosesenkende Therapie bei Herzinsuffizienz

### *Herzinsuffizienz mit erhaltener Linksventrikelfunktion LVEF ≥* *50* *% (HFpEF*)

Die HFpEF betrifft etwa 50 % der Patient:innen, welche gleichzeitig an Diabetes mellitus und Herzinsuffizienz leiden [30]. Entsprechend den aktuell gültigen Leitlinien der ESC sollte bei vorliegenden klinischen Beschwerden (Atemnot, Orthopnoe, Beinödemen, nächtliche Atemnot, reduzierte Belastbarkeit, Müdigkeit) fortführend eine BNP- bzw. NT-proBNP Bestimmung gefolgt von einer Echokardiographie durchgeführt werden [31].

Mittlerweile liegen für Empagliflozin als auch für Dapagliflozin positive Daten zur Behandlung der HFpEF vor.

Die Emperor-Preserved Studie untersuchte 5988 Patient:innen, deren linksventrikuläre Auswurffraktion > 40 % war und stellt die erste Studie eines Gliflozins in diesem Kollektiv dar.

Letztlich bewirkte Empagliflozin eine signifikante Reduktion des primären Endpunktes (kardiovaskulärer Tod oder Hospitalisation aufgrund von Herzinsuffizienz) um 21 % (HR 0,79, 95 % CI 0,69–0,90) [32].

Analog zur Emperor Preserved Studie zeigte sich für Dapagliflozin in der DELIVER-Studie ebenfalls ein positiver Effekt bei Patient:innen mit einer LVEF > 40 %. Über einen Nachbeobachtungszeitraum von 2,3 Jahren konnte der primäre Endpunkt (Verschlechterung der Herzinsuffizienz oder kardiovaskulärer Tod) signifikant reduziert werden (HR 0,82 CI 0,73–0,92; *p* < 0,001) [33].

Zusammenfassend legen diese Resultate nahe, dass bei Vorliegen einer HFpEF eine Therapie mit Dapagliflozin oder Empagliflozin unabhängig vom HbA_1c_ etabliert werden soll.

### *Herzinsuffizienz mit reduzierter Linksventrikelfunktion LVEF ≤* *50* *% (HFrEF*)

Dapagliflozin, Empagliflozin, als auch Canagliflozin haben in den jeweiligen Endpunktstudien deutliche Reduktionen in der Hospitalisierung aufgrund einer Herzinsuffizienz gezeigt.

In der DAPA-HF Studie wurden Personen mit vorbestehender Herzinsuffizienz mit reduzierter Auswurffraktion eingeschlossen (LVEF < 40 % und Symptome entsprechend New York Heart Association (NYHA) Klasse II–IV und erhöhte N‑terminal pro-B-type natriuretic peptide-Spiegel). Ein vorbestehender Diabetes mellitus Typ 2 war kein verpflichtendes Einschlusskriterium. In der Dapagliflozin-therapierten Gruppe zeigte sich eine signifikante Reduktion des primären Endpunktes (Verschlechterung der Herzinsuffizienz – dies war entweder eine Hospitalisierung oder dringliche Visite mit intravenöser Herzinsuffizienztherapie – oder kardiovaskulärer Tod) (HR 0,74; 95 % CI 0,65–0,85; *p* < 0,01). Dieser Effekt war unabhängig davon, ob ein Diabetes mellitus Typ 2 vorbestehend war oder nicht [32]. Neben der DAPA-HF-Studie liegen auch die Resultate der EMPEROR-Reduced-Studie, welche mit Empagliflozin durchgeführt wurde, vor. Insgesamt wurden 3730 Patient:innen, die an einer manifesten Herzinsuffizienz NYHA II–IV erkrankt waren und eine LVEF ≤ 40 % hatten untersucht. Im Rahmen dieser randomisiert kontrollierten Studie wurde Empagliflozin 10 mg mit Placebo zusätzlich zur etablierten, leitliniengerechten Therapie untersucht. Die mediane Nachbeobachtungszeit lag bei 16 Monaten, die Prävalenz des Diabetes mellitus lag bei 49,8 %. Unabhängig vom Vorliegen eines Diabetes mellitus konnte der primäre Endpunkt (Hospitalisierungsrate aufgrund von Herzinsuffizienz oder kardiovaskulärer Tod) durch die Gabe von Empagliflozin signifikant gesenkt werden (HR 0,75; 95 % CI: 0,65–0,86; *p* < 0,001) [34]. Entgegen den Resultaten von DAPA-HF konnte in der EMPEROR-Studie keine signifikante Reduktion des kardiovaskulären Todes als seperatem Endpunkt dokumentiert werden. Im direkten Vergleich lag die Ereignisrate für den primären Endpunkt in der EMPEROR-Studie höher als in DAPA-HF, was sich letztlich auch durch die Tatsache erklären lässt, dass die Patient:innen in der EMPEROR-Studie insgesamt fortgeschrittenere Stadien der Herzinsuffizienz aufwiesen.

### Weitere Medikamentenklassen

In retrospektiven Datenanalysen konnte bei mit Metformin behandelten herzinsuffizienten Patient:innen eine signifikant geringere Mortalität nachgewiesen werden [35, 36]. Dennoch muss in Betracht gezogen werden, dass die hämodynamisch instabile Herzinsuffizienz einen signifikanten Risikofaktor für die potenziell letale Laktatazidose darstellt, welche jedoch mit etwa 9 Fällen pro 100.000 Personenjahren auftritt.

Pioglitazon ist laut Zulassungstext bei Herzinsuffizienz NYHA I–IV aufgrund des Risikos einer gesteigerten Flüssigkeitseinlagerung kontraindiziert und darf daher bei manifester Herzinsuffizienz generell nicht eingesetzt werden.

In den kardiovaskulären Endpunktstudien der DPP-IV Hemmer wurde in der SAVOR-TIMI 53 Studie ein 27 % höheres Risiko für die Hospitalisation aufgrund einer Herzinsuffizienz unter Saxagliptin beobachtet. In EXAMINE (Alogliptin), CARMELINA (Linagliptin) und TECOS (Sitagliptin) konnte diese Assoziation jedoch nicht beobachtet werden [37].

Hinsichtlich der GLP‑1 Analoga haben sich mögliche positive Effekte bei Patient:innen mit chronischer Herzinsuffizienz in den einzelnen CVOTs nicht bestätigt.

Liraglutid zeigte in der LEADER – Studie keine Reduktion von Herzinsuffizienz Endpunkten, es scheint was diese anbelangt also neutral zu sein.

## Interessenkonflikt

M. Clodi hat von folgenden Unternehmen, die auch fördernde Mitglieder der ÖDG sind, Forschungsunterstützungen und/oder Honorare erhalten: Abbott Diabetes Care, AstraZeneca, Boehringer Ingelheim, Daiichi-Sankyo, Dexcom, Eli Lilly, Medtronic, MSD, Novartis, Novo Nordisk, Sanofi. C.H. Saely hat von folgenden Unternehmen, die auch tlw. fördernde Mitglieder der ÖDG sind, Forschungsunterstützungen und/oder Honorare erhalten: Sprecher- und Berater-Honorare von Astra Zeneca, Böhringer-Ingelheim, Sanofi Aventis, Lilly, Merck, MSD. F. Hoppichler ist als Referent für folgende Firmen tätig: Boehringer Ingelheim RCV GmbH & Co KG, Sanofi-Aventis GmbH, Daiichi Sankyo Austria GmbH, AstraZeneca Österreich GmbH, Amgen GmbH, Novo Nordisk Pharma GmbH, Eli Lilly Gesellschaft m.b.H. M. Resl hat von folgenden Unternehmen, die teils auch fördernde Mitglieder der ÖDG sind, Honorare für Vorträge und/oder Beratertätigkeit bzw. Fortbildungsunterstützung erhalten Boehringer Ingelheim, Mylan, Dexcom, Abbott. C. Steinwender gibt an, dass kein Interessenskonflikt besteht. H. Stingl legt offen, dass er von folgenden Unternehmen, die teils auch fördernde Mitglieder der ÖDG sind, Forschungsunterstützungen, Honorare für Vorträge und/oder Beratertätigkeit, bzw. Fortbildungsunterstützung im Rahmen der (dienst)rechtlichen Rahmenbedingungen (Einladung zu Kongressen) erhalten hat: Abbott, Amgen, AstraZeneka, Boehringer Ingelheim, Daiichi Sankyo, Eli Lilly, Menarini, MSD, Novo Nordisk, Novartis, Sanofi Aventis, Servier, Viatris. Zusätzlich gibt H. Stingl an, dass für ihn persönlich kein Interessenkonflikt vorliegt. T.C. Wascher: Forschungsunterstützung, Honorare für Beratung oder Fortbildung von Boehringer Ingelheim, Astra Zeneca, Ely Lilly, Novo Nordisk, Bayer. Y. Winhofer hat von folgenden Unternehmen, die auch tlw. fördernde Mitglieder der ÖDG sind, Forschungsunterstützungen und/oder Honorare erhalten: Abbott, Amgen, Astra Zeneca, Boehringer Ingelheim, Daiichi Sankyo, Eli Lilly, Medtronic, MSD, Novartis, Novo Nordisk, Roche, Sanofi Aventis. H. Sourij hat von folgenden Unternehmen, die auch tlw. fördernde Mitglieder der ÖDG sind, Forschungsunterstützungen (an die MedUni Graz) und/oder Honorare erhalten: Amarin, Amgen, AstraZeneca, Boehringer-Ingelheim, Bayer, Daiichi Sankyo, Eli Lilly, Kapsch, Merck Sharp & Dohme, Novo Nordisk, Sanofi-Aventis.
